# Cannabinoid receptor 1 activity promotes microbial dysbiosis by stimulating the oxygen-dependent expansion of Enterobacteriaceae

**DOI:** 10.64898/2026.10.10.758071

**Published:** 2026-10-12

**Authors:** Mary K. Mitchell, Mikayla A. Carty, Nayeli L. Snow, Mary T. Archambault, Addison E. Budsberg, Elisa V. Carrillo, Emily E. Vignieri, Ethan A. Older, Olivia L. Rometo, Lauren E. Van de Wal, Kayla J. Floyd, Lindsey R. Baker, Claire A. Kunkle, Jie Li, Melissa Ellermann

## Abstract

The endocannabinoid system is comprised of bioactive lipids that signal via cannabinoid receptors, which can modulate barrier and immune functions along the gastrointestinal tract. Clinical studies indicate that endocannabinoid activity is dysregulated in inflammatory bowel diseases. Moreover, an increase in gut endocannabinoids is correlated with microbial dysbiosis in Crohn’s disease. Here, we show that endocannabinoid activity promotes metabolic dysfunction within the colonic mucosa, resulting in microbial dysbiosis and exacerbated colitis. Stimulation of the cannabinoid receptor CB1 resulted in the expansion of Enterobacteriaceae within the gut microbiome, a common feature of inflammation-associated dysbiosis. Preceding this microbial change, CB1 activity induced transcriptional signatures of mitochondrial stress and immune activation within the colonic mucosa. This was followed by a reduction in mucosal ATP production and the induction of aerobic respiration genes in luminal *Escherichia coli* . Genetic inactivation of aerobic respiration in *E. coli* limited its proliferation, indicating that CB1 activity stimulates oxygen-dependent Enterobacteriaceae growth. In IBD susceptible mice, endocannabinoid activity promoted mucosal infection by an *E. coli* pathobiont, which corresponded with elevated pro-inflammatory markers. In inflammation resistant mice, acute CB1 stimulation was sufficient to accelerate the infection dynamics of an Enterobacteriaceae pathogen, resulting in exacerbated colitis. Taken together, our data support a model in which endocannabinoid signaling at the CB1 receptor disrupts respiratory metabolism in the colon, compromising the anoxic environment of the lumen that is critical for maintaining aerobically respiring pathogens at low abundance. More broadly, our findings highlight a proinflammatory effect of endocannabinoid activity that can contribute to intestinal disease development.

